# Supplementary material for: Lessons learnt for digital inclusion in underserved communities from implementing a covid virtual ward
Source: PLOS Digit Health. 2022 Nov 16;1(11):e0000146. doi: 10.1371/journal.pdig.0000146 (PMC9931265; doi:10.1371/journal.pdig.0000146)
Supplement: S1 Index — (DOCX) [file pdig.0000146.s001.docx]

**S1 Index:**

1. You were given enough information prior to discharge about the rationale of the virtual ward round (Strongly agree (1), agree (2) , neutral (3), disagree (4), strongly disagree (5))
2. You were given enough information prior to discharge about how to interpret your saturation results
3. You were given enough information about when to contact a healthcare professional about your results
4. You were given enough information about how to use the pulse oximeter
5. Going home with a pulse oximeter made you feel more reassured then going home without one
6. Going home with a pulse oximeter made your family feel more reassured than going home without one
7. Having a pulse oximeter made you feel more anxious during your recovery (what I want to get across in this question is whether they think they noticed symptoms more)
8. Having a pulse oximeter made your family feel more anxious during your recovery
9. You felt as though your results were being monitored and looked at by a healthcare professional
10. You were contacted enough about your results and recovery
11. The pulse oximeter affected your decision about whether to come back to hospital or not
12. It was reassuring to have a contact number for someone to talk to
13. The app was easy to use
14. Overall comments
