# Supplementary material for: Lessons learnt for digital inclusion in underserved communities from implementing a covid virtual ward
Source: PLOS Digit Health. 2022 Nov 16;1(11):e0000146. doi: 10.1371/journal.pdig.0000146 (PMC9931265; doi:10.1371/journal.pdig.0000146)
Supplement: S2 Index — (DOCX) [file pdig.0000146.s002.docx]

**S2 Index:**

Data was collected for patients that **did not use** or register for the app via the following questions:

1. Clarify age and ethnicity
2. You were given enough information prior to discharging about using the pulse oximeter?
3. If no – what other information did you need?
4. You were given enough information about how to download and register with the Huma app.
5. If no – what other information did you need?
6. You were given enough information about how to input your results onto the app.
7. If no – what other information did you need?
8. You have access to the equipment to allow this to happen
9. Phone?
10. Internet?
11. The app was well designed and easy to use
12. Do you think training would have helped?
13. Do you have any disability affecting your ability to use apps?
14. Would an app in a different language have helped?
15. Did you have any problems related to:
16. Huma app
17. Pulse oximeter
18. Access
