# Supplementary material for: Lessons learnt for digital inclusion in underserved communities from implementing a covid virtual ward
Source: PLOS Digit Health. 2022 Nov 16;1(11):e0000146. doi: 10.1371/journal.pdig.0000146 (PMC9931265; doi:10.1371/journal.pdig.0000146)
Supplement: Attachment — Submitted filename: Revisions Plos Digital Health - responses to reviewers.docx [file pdig.0000146.s004.docx]

Dear Editorial Office,

We would like to thank both reviewed for their time and comments to improve our manuscript. Below is a point by point response:

**Reviewer #1:**

- Important methodological details are missing: What instrument did you use (a survey, a survey + interviews, a survey + room for open comments, survey + data)?

**Many thanks. This has now been clarified in methodology.**

- Provide details on ethics approval and informed consent for data collection, data use and analysis. How did you get age-related info from the app users if you did not ask that in Index1? Did you have authorisation to use such information, say, from health records for research purposes?

**Many thanks. This was registered as a service improvement project . Our Trust process is that feedback from users of a service or those referred to a service can also be collected as part of this process.**

- Since you did not ask app users about their ethnicity, results in figure 2 are not really informative.

**Thank you. We have removed this table and have referenced this limitation in the results. Unforunately, app users were not asked their ethnicity so we do not have this data. This only way to do this would be to back to cerner (trust online health records).**

- How did you analyse data form open comments in Index1? (see table 2)

**This data was not used for formal analysis.**

- Is question 9 in Index2 an open comment question or a y/n question?

**Y/N**

- How comes you did not ask about socio-educational status, given you rightly state this is a major determinant of digital exclusion? In the absence of this more granular information it is hard to make sense of, for instance, answers regarding ease-of-use.

- How comes proficiency in English, access to and ability to use apps are inclusion criteria, and some respondents ended up not being able/willing to use the app and having problems with English? This is hard to make sense of.

**This is now referred to in the discussion re: inappropriate referrals to the CVW and study limitations.**

- Question 5.c in Index2 seems a y/n question, while question 13 in Index 1 (on the same topic) uses a scale. It is thus hard to interpret the results in this case.

**Many thanks, and we agree. We have adapted the results sections and removed a lot of the graphical results representation to avoid this confusion.**

- Important limitations to specify in the paper: the authors need to specify that the questionnaires they have used are not validated instruments to assess users acceptance of health-related digital technologies.
- Furthermore, the questions are framed in such a way that they capture patients subjective impressions, rather than being a reliable measure of, e.g., whether or not enough information was provided or training was needed.

**Many thanks. We have now discussed the study limitations in the discussion section, including your points above.**

- Discussion: this section is not actually a discussion, but rather a presentation of other results (that should go in the results section instead) followed by commentary. I'd expect this section to discuss the results in light of similar studies and relevant literature in the the field of digital health and digital health ethics.

**Many thanks. The results and discussion sections have been edited accordingly.**

Other minor points:
- I wouldn't say "in both surges" in the abstract as there have been more than two waves of COVID19 in the UK. Add "in the timeframe of the study" or something of the like.
- Did CVW admit 142 or 143 patients? See lines 53 and 120.
- I would not use the expressions "digitally included" and "digitally excluded" as they carry an evaluative connotation. Use "app users" and "app non-user" instead.

**Many thanks. Changes made as suggested. The CVW admitted 142 patients. Line 57 (previously 52) has been edited to reflect this correction.**

**Reviewer #2:**
I wonder if the title could be changed to highlight the novel aspects of the study. For example: “Lessons learnt for digital inclusion in marginalised/underserved communities (use most appropriate term here and keep consistent throughout the write up) from implementing a covid virtual ward”

**Many thanks, the title has been changed as suggested.**

Abstract:
•       Objective: I would recommend changing “causing” to “that lead to” or “factors associated with” or “drivers of” causality not determined via your study, rather associations and differences noted. Another way to re-work the objectives would be to replace “factors causing digital exclusion in patients” could be changed to “facilitators and barriers associated with uptake of remove digital monitoring in the outpatient setting (or at home) during covid-19 pandemic in economically deprived and/or ethnically diverse groups”

**Many thanks. This now reads “factors associated with”.**

Intro:
Overall, this section is well written and introduces key issues in a concise manner. I would recommend the following minor edits
•       Line 35: economically “deprived”
•       Line 48 replace “forced” with “required to”
•       Line 70, specify with groups
•       Line 68-69, give examples of remote/digital monitoring in the outpatient setting
•       Line 80 – what do you mean by vulnerable groups? Are you referring to underserved communities or economically deprived and/or ethnically diverse groups? Might be worth ensuring that terminology is kept consistent throughout the paper
•       Line 77-78 – revise objective of the study as suggested above.

**Many thanks. Line 37 (previous 35) and 51 (previously 48) changed as suggested. Examples of groups affected now given lines 74-76.**

**Examples of digital monitoring systems lines 70-71 included with references updated.**

Method:
A few further details required for clarity.
•       Line 85-86 – how many patients were assessed for eligibility and how many were finally enrolled in the study.

**Thank you, this is now clarified in the results section. All patients who discharged from the CWV were eligible (97 app users, 65 non-app users). We have clarified those that were unreachable or refused to take part.**

•       By design the eligibility criteria excludes some groups – such as those unable to speak English or having a carer/family member who speaks English or have access to a smart phone or be willing to download the Huma app. Was data collected on how many were excluded based on these criteria? It might also be worth reflecting on this in the discussion.
•       Line 95: were other methods considered/used to refer patents into CVW– for example telephone rather than the exclusive use of emails to refer patients to the CVW? It might be worth also reflecting on whether the method of enrolling into the study itself might have been digitally biased and excluded those who might experience barriers to accessing digital technologies.

**Many thanks. Patients were referred by other healthcare professionals via email to enable a 24 hour referral service. The issue is more, inappropriately referred patients, which we have now referenced in the discussion.**

•       Line 111: was feedback collected as a survey? Who conducted this? How was feedback collected? Online, via the app, on the phone? Were questions open ended, closed ended, some example questions – more details required here.
•       Lines 114-117: how were classifications made on whether users of CVW were “app users” or “non app users”.  Who made this classification, how and when was it done? I thought having the HUMA app was an inclusion criterion (see Line 94). My understanding then is that “non app users” are patents who had access/had downloaded app but did not use the app.

**Many thanks. This is now clarified in the text.**

Results:
This section needs work. I would suggest having clear research questions and then reorganising your result to include means, and appropriate statistical tests to show significant differences between the groups and highlight any interesting results.

•       Table 1 and Figure 1 do not contribute to the understanding of the topic or add value to the result section.

**They have been removed.**

•       I would recommend starting with a section describing the participants characteristics of both groups– including ethnic origin of participants (Figure 2).
•       I would then present the results as means (SDs) and then move on to do appropriate test (t-tests) to show difference between groups.
•       Figure 4 results interesting and most relevant. However no real reporting of the results in the text. Can this be elaborated and some more appropriate tests conducted.

**Many thanks. This section has been re-written and additional statistical analysis included. Unfortunately, given the questions asked to each cohort we are unable to statistically compare like with like in many cases (Referred to in discussion). All tables with no clear addition to text have now been removed.**


Discussion:
•       This section reads more as a summary of the results. I would have liked to see some further engagement with the wider literature in the field. There is potential here to highlight the implications this work has on the lessons for digital inclusion in underserved/marginalised communities.
•       Could this be added to this section. Have other studies evaluated CVW and digital exclusion? Or can the current results be compared with wider studies on digital exclusion?
•       What contributions does the current research make to the field?
•       What were some limitations of the current study?
•       Are there any further lessons that can be learnt for implementation in practice?
•       Do the authors have any suggestions for future studies in the area?

**Many thanks. The discussion has now been re-written with the old discussion largely lying within results now. We have discussed our study limitations, further scope for research and how we have added to the research base with our work.**

**In terms of relevant literature, we have found nothing particularly relevant as there is very little on digital exclusion in the healthcare setting (other than those papers already referenced) and nothing on digital exclusion in the covid virtual ward setting, if the reviewers are aware of papers please let us know.**
